# mTORC1 regulates mannose-6-phosphate receptor transport and T-cell vulnerability to regulatory T cells by controlling kinesin KIF13A

**DOI:** 10.1038/celldisc.2017.11

**Published:** 2017-04-25

**Authors:** Khawaja Ashfaque Ahmed, Jim Xiang

**Affiliations:** 1 Cancer Research, Saskatchewan Cancer Agency, Saskatoon, Saskatchewan, Canada; 2 Department of Oncology, College of Medicine, University of Saskatchewan, Saskatoon, Saskatchewan, Canada

**Keywords:** M6PR, T-cell fate, mTORC1, KIF13A, T_reg_ cell, apoptosis

## Abstract

Mannose-6-phosphate receptor (M6PR) that facilitates cellular uptake of M6P-bearing proteins, including serine-protease granzyme-B (Gzm-B) has an important role in T-cell activation, migration and contraction. However, molecular mechanisms controlling M6PR expression in T cells remain poorly understood. Here, we show that M6PR expression on T cells is distinctively controlled by two common γ-chain cytokines interleukin-2 (IL-2) and IL-7, and the differential M6PR expression is not caused by an altered synthesis of M6PR protein, but is a result of distinct regulation of kinesin-3 motor-protein KIF13A that transport M6PR onto cell surfaces. Using signaling pathway-specific inhibitors, we determine that IL-2 and IL-7 distinctly regulate KIF13A and β1-adaptin and cell-surface M6PR by controlling a kinase mammalian target of rapamycin complex-1 (mTORC1). Inflammatory cytokine IL-2 and prosurvival cytokine IL-7 induce strong and weak activation of mTORC1, leading to up- and downregulation of motor-protein KIF13A and KIF13A-motorized M6PR on T cells, and formation of IL-2 and IL-7 effectors with M6PR^high^ and M6PR^low^ cell-surface expression, respectively. Inhibition of mTORC1 by rapamycin reduces T-cell expression of KIF13A and cell-surface M6PR, and increases T-cell survival in *Listeria monocytogenes-*infected mice. Using regulatory T (T_reg_)-cell-enriched mouse tumor model, we determine that M6PR^high^ IL-2 effectors but not M6PR^low^ IL-7 effectors adoptively transferred into tumors are vulnerable to T_reg_ Gzm-B-mediated cell apoptosis. Inhibition of mTORC1 or small interfering RNA-mediated knockdown of KIF13A or M6PR renders IL-2 effectors refractory to T_reg_ Gzm-B lethal hit. Overall, our data offer novel mechanistic insights into T-cell M6PR regulation, and T_reg_-resistant/T_reg_-susceptible phenomenon. Furthermore, regulation of T-cell fate vis-à-vis T_reg_ suppression via the mTORC1-KIF13A-M6PR axis provides a proof of concept for therapeutic strategies to target cancer, infectious and autoimmune diseases.

## Introduction

CD8^+^ T cells provide protection against pathogens and cancer [1, 2]. In response to an infection, pathogen-specific CD8^+^ T cells undergo rapid expansion, followed by a contraction phase, in which 90–95% of effector CD8^+^ T cells die of cell apoptosis that likely serves to limit immunopathology by controlling the duration of T-cell responses [1–3]. The remaining 5–10% of effector CD8^+^ T cells survive the contraction, and further differentiate into long-lived memory T cells [1–3]. Despite major advances in our understanding of T-cell immunity, the issue of T-cell fate decisions remains enigmatic [1, 2, 4–6]. Aberrant T-cell contraction can lead to diseases such as autoimmunity and immunodeficiency [2, 7, 8]. It is well recognized that host immune responses to pathogens and host immunity to cancer can be improved by preventing T_reg_-mediated suppression of T-cell effectors [9, 10]. Alternatively, enhancement of effector T cell’s susceptibility to T_reg_-mediated suppression may be effective in autoimmunity [11–13]. Deciphering the signals that determine which T cells die and which ones survive during contraction and T_reg_ suppression will help in regulating the number of T cells seeding the memory T-cell pool and developing strategies for vaccine design and immunotherapy for cancer and various diseases [2, 8, 11, 12, 14].

Mannose-6-phosphate receptor (M6PR) is a versatile receptor [15], which is involved in several biological process including lysozyme biogenesis [16] and granzyme-B (Gzm-B) uptake [17]. It also acts as a sink for insulin-like growth factor-II [18, 19] and promotes activation of the latent form of transforming growth factor-β [20]. M6PR is upregulated on activated T cells that possibly facilitates T-cell entry of inflammatory sites [21], and involved in T-cell activation through recruitment of lymphocyte-specific protein tyrosine kinase to the immunological synapse [22]. Moreover, we recently demonstrated a novel role for the M6PR in determining T-cell fate decisions during the contraction phase in *Listeria monocytogenes*-infected mice [3, 23]. We found that nearly all antigen-specific CD8^+^ T cells upregulated M6PR during the early phase of *Listeria monocytogenes* infection, and subsequently ~25% of them downregulated M6PR at the peak [3]. Effector T cells with high M6PR expression (M6PR^high^) revealed susceptibility to CD4^+^CD25^+^FoxP3^+^ regulatory T (T_reg_) cell’s Gzm-B-mediated apoptosis, whereas those with low M6PR expression (M6PR^low^) preferentially escaped apoptosis and contraction [3], indicating a critical role for M6PR in dictating life and death decisions in CD8^+^ T cells. A recent study reported that M6PR expression on T cells of untreated HIV-1-infected patients is significantly higher than healthy human controls, suggesting that perturbed T-cell memory compartment in HIV-1 patients may be associated with increased susceptibility of these T cells to Gzm-B-mediated cell apoptosis [24]. M6PR may thus represent a double-edged sword controlling both proliferation [22] and attrition [3, 23, 24] in T cells. Therefore, understanding the signals that regulate M6PR expression in T cells will have implication for modulating T-cell immunity in both infectious and autoimmune diseases [23, 24].

Signals from common receptor γ-chain (γ_c_) family of cytokines greatly influence life versus death decisions in CD8^+^ T cells [8, 25]. Interleukin-2 (IL-2) and IL-7 are the two best-studied γ_c_ family of cytokines that dictate different T-cell fates even though they initiate similar signaling cascades [8, 25], and upregulate antiapoptotic proteins of the Bcl-2 family [25]. IL-2 signaling leads to activation-induced cell death of CD8^+^ T cells [26, 27]. In contrast, IL-7 promotes CD8^+^ T-cell survival and memory formation [28]. Notably, previous studies report that T_reg_ cells preferentially kill IL-2-stimulated T cells [29] but not IL-7-stimulated T cells [30]. However, the underlying mechanisms are unclear. Interestingly, in our recent study, we observed that T_reg_ cells preferentially kill M6PR^high^ but not M6PR^low^ CD8^+^ T cells in *L. monocytogenes* infection [3]. Thus, our recent observation provides a useful platform to study a potential link between these two observations with respect to effector’s susceptibility or refractoriness to T_reg_-mediated suppression and to elucidate the molecular mechanism for regulation of M6PR expression in T cells and distinct vulnerability of IL-2 and IL-7 effectors to T_reg_ suppression.

In this study, we *in vitro* generated IL-2 and IL-7 effectors derived from congenic mice and assessed vulnerability of IL-2 and IL-7 effectors to T_reg_ cells *in vitro*, and then *in vivo* in a mouse model of subcutaneous tumor, B16 melanoma that provides an *in vivo* T_reg_-cell-enriched environment. We demonstrate that IL-2 but not IL-7 renders T-cell effectors susceptible to Gzm-B-mediated killing by enhancing cell-surface M6PR expression through an upregulation of kinesin-3 motor-protein, KIF13A, which transports M6PR onto the cell surfaces. We further identify that a distinct signal strength of mammalian target of rapamycin complex-1 (mTORC1) kinase induced by IL-2 and IL-7 differentially controls KIF13A-transported cell-surface M6PR display, eventually determining the vulnerability of T cells to T_reg_ Gzm-B uptake-induced T-cell death and leading to distinct T-cell fates [15, 17].

## Results

### IL-2 but not IL-7 upregulates M6PR-rendering effector T cells vulnerable to T_reg_-derived Gzm-B lethal-hit *in vitro*

**Figure 1:**
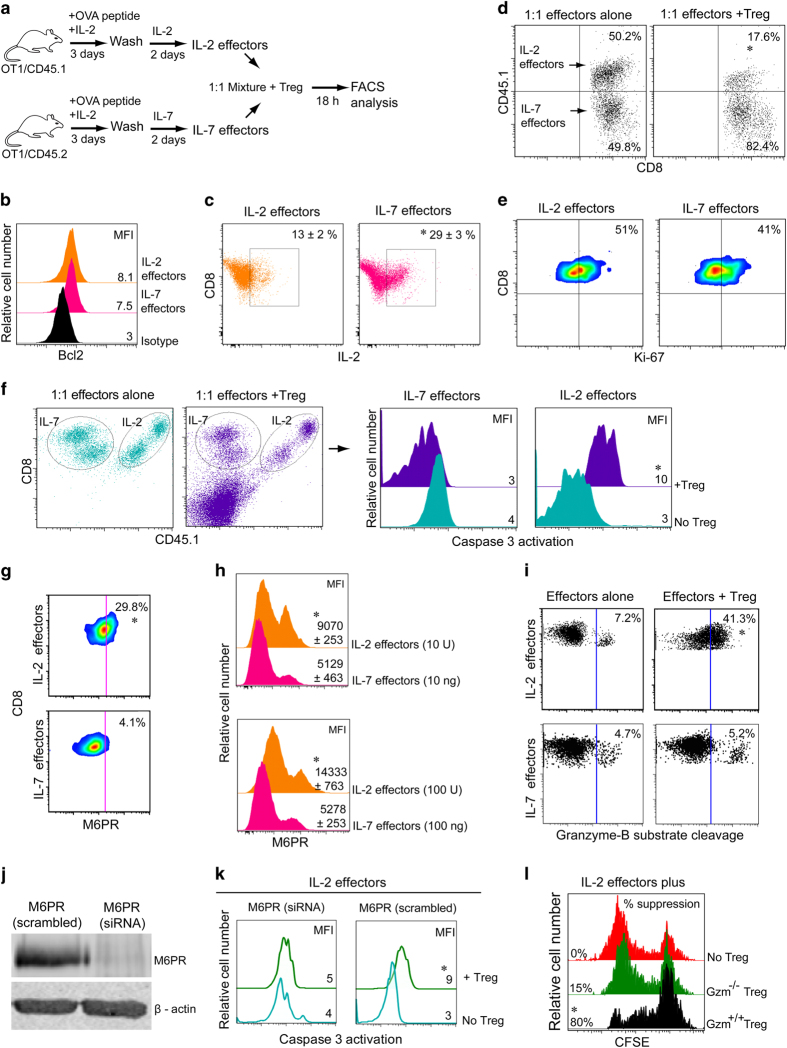
IL-2 and IL-7 differentially regulates T-cell M6PR dictating distinct T-cell vulnerability to T_reg_-mediated suppression. (**a**) OVAI peptide (0.1 nM) and IL-2 (100 U ml^−1^) activated OT-I CD8^+^ cells (CD45.1 or CD45.2 congenic), subsequently cultured in IL-2 (100 U ml^−1^) or IL-7 (10 ng ml^−1^) for 2 days to generate IL-2(CD45.1) and IL-7(CD45.2) effectors, respectively. (**b**) IL-2 and IL-7 effectors revealed comparable Bcl-2, (**c**) higher intracellular IL-2 in IL-7 effectors. (**d**–**f**) The 1:1 mixture of IL-2(CD45.1) and IL-7(CD45.2) effectors cocultured with or without T_reg_ cells (1:4), (**d**) ratios of surviving IL-2:IL-7 effectors (*n*=6) after 18 h, (**e**) effectors revealed comparable Ki-67 expression and (**f**) distinct caspase activation after 1 h of incubation in the presence of a cell-permeable fluorogenic caspase-3 substrate, as detected by flow cytometry. (**g**) IL-2 and IL-7 effectors revealed divergent cell-surface M6PR expression. (**h**) Comparison of M6PR expression in IL-2 and IL-7 effectors by signal strength using two 10-fold different cytokine doses, as indicated in the figure. (**i**) Coculturing for 30 min with or without T_reg_ cells in media containing a fluorogenic Gzm-B substrate demonstrated Gzm-B activation in IL-2 effectors but not in IL-7 effectors. (**j**) siRNA-mediated knockdown of M6PR in IL-2 effectors. (**k**) M6PR knockdown prevented Gzm-B entry as evidenced by the abrogation of caspase-3 activation in IL-2 effectors. (**l**) GzmA^−/^^−^B^−/−^ T_reg_ failed to prevent the proliferation of CFSE-labeled CD8^+^ T cells during coculturing with CD3/CD28 beads plus IL-2. *P< 0.05, versus cohorts of IL-2 effectors without T_reg_ cells. Data are representative of two or three independent experiments. MFI, mean fluorescence intensity.

We first purified naïve CD8^+^ T cells derived from ovalbumin (OVA)-specific T-cell receptor (TCR) transgenic OT-I (CD45.1 or CD45.2 congenic) mice [1], and then stimulated them *in vitro* with OVA peptide (OVA_257–264_, SIINFEKL) plus IL-2 for 3 days, followed by another 2 days of culturing them in either IL-7 or IL-2 (Figure 1a) [31]. Such cytokine-activated IL-2 and IL-7 effectors showed similar levels of the antiapoptotic protein Bcl-2 (Figure 1b), but intracellular IL-2 was significantly higher in IL-7 effectors (Figure 1c). Higher intracellular IL-2 in IL-7 effectors is in agreement with previous reports [32]. Decreased intracellular IL-2 in IL-2 effectors is probably due to the negative feedback mechanism as reported previously [33, 34]. Cell-surface expression of CD44, CD25 and CD127 was more in IL-2 effectors, whereas CD62L and CXCR3 were higher in IL-7 effectors (Supplementary Figure S1). Unlike previous studies [29, 30, 35], here we developed a unique *in vitro* culture system providing the same quality and quantity of T_reg_ cells, and coculture media to assess the distinct vulnerability of cytokine-stimulated effector T cells to T_reg_ suppression, wherein 1:1 mixture of CD45.1 or CD45.2 congenic IL-2 and IL-7 effectors was cocultured with T_reg_ cells for another 18 h in medium plus IL-2 (Figure 1a), thus providing the same culturing environment to these effectors. Effectors were also adoptively injected into recipient mice that previously received T_reg_ cells (Supplementary Figure S2) to observe the vulnerability of these effector T cells to T_reg_ suppression *in vivo*. In agreement with previous reports [29, 30], we recovered significantly fewer IL-2 effectors than IL-7 effectors (Figure 1d and Supplementary Figure S2). Cell-surface expression levels of CXCR3 in IL-2 and IL-7 effectors (Supplementary Figure S1) suggest that differential migration may not be the real reason for less number of IL-2 effectors in splenocytes. Because IL-2 and IL-7 effectors cocultured with T_reg_ cells had comparable Ki-67 (a proliferation marker) staining (Figure 1e), our data thus suggest that an increased cell death but not a proliferation defect contributes to the attrition of IL-2 effectors. To further confirm this possibility, 1:1 mixture of congenic IL-2(CD45.1) and IL-7(CD45.2) effectors was cocultured with or without T_reg_ cells (1:4) for 1 h at 37 °C in the presence of PhiPhiLux-G2D2, a cell-permeable fluorogenic caspase-3 substrate (DEVDGI), following the kit protocol (OncoImmunin, Gaithersburg, MD, USA). When the target cells undergo apoptosis, the substrate is cleaved by caspase-3, resulting in emission of fluorescent light. Our flow cytometry analysis clearly demonstrated more caspase-3 activation in IL-2 effectors than in IL-7 effectors, suggesting preferential cell apoptosis in IL-2 effectors (Figure 1f).

Cell-surface M6PR (Gzm-B receptor) has an important role in Gzm-B-mediated cell apoptosis [15, 17], and our recent finding showed that M6PR^high^ effectors are more susceptible to T_reg_-derived Gzm-B lethal hit [3]. Therefore, we anticipated that cytokine signals would have differentially regulated cell-surface M6PR expression in T cells. Interestingly, IL-2 stimulation generated M6PR^high^ effectors, whereas IL-7 induced M6PR^low^ effectors, as evidenced by flow cytometry analysis (Figure 1g). To rule out the possibility that differential M6PR expression in IL-2 and IL-7 effectors is due to the different cytokine doses, we increased the concentration of IL-7 from 10 to 100 ng ml^−1^ and decreased IL-2 concentration from 100 to 10 U ml^−1^. Interestingly, compared with IL-7 effectors, IL-2 effectors had significantly higher cell-surface M6PR expression in both the conditions (Figure 1h), suggesting that IL-7-induced M6PR^low^ effectors was not due to the dose of cytokines. We next examined a possibility of the differential uptake of Gzm-B by IL-2 and IL-7 effectors *in vitro*. To test this issue, we fluorescently labeled IL-2 or IL-7 effectors, and then cocultured them with T_reg_ cells (1:4) for 30–40 min at 37 °C in the presence of GranToxiLux, a cell-permeable fluorogenic substrate of Gzm-B. Flow cytometry demonstrated GranToxiLux cleavage in ∼41% vs ∼5% of IL-2 vs IL-7 effectors, respectively (Figure 1i). To further confirm that M6PR^high^ predisposes IL-2 effectors to Gzm-B lethal hit, we knocked down M6PR using pooled insulin-like growth factor 2R (M6PR) small interfering RNA (siRNA) lentivirus (piLenti-siRNA-GFP) (Figure 1j). IL-2 effectors transfected with M6PR (scrambled) or M6PR (siRNA) were cocultured with T_reg_ cells (1:4) for 1 h at 37 °C in the presence of PhiPhiLux-G2D2. Flow cytometry analysis revealed caspase-3 activation in M6PR (scrambled)-transfected effectors but not in M6PR (siRNA)-transfected effectors, confirming a link between M6PR expression and Gzm-B uptake (Figure 1k).

We and others have shown that the Gzm-B-perforin pathway critically regulated T_reg_-mediated killing of CD8^+^ T cells *in vitro* [3, 29] and *in vivo* [3, 9, 10, 36, 37]. To confirm that Gzm-B-mediated cell death of effectors is derived from T_reg_ cells but not from effectors themselves, we performed another set of *in vitro* experiments. Effectors were labeled with carboxyfluorescein succinimidylester (CFSE, 3 μm), and cultured alone or with T_reg_ cells derived from wild-type (WT) or Gzm-A^−/^^−^B^−/^^−^ mice, and then stimulated with anti-CD3/CD28 Ab-coated latex beads (MACS) plus IL-2 (100 U ml^−1^) for 5 days. Flow cytometry analysis revealed that Gzm-B-sufficient T_reg_ cells killed 80% of proliferated IL-2 effectors (Figure 1l), whereas Gzm-B-deficient T_reg_ cells failed in killing IL-2 effectors as evidenced by CFSE dilution similar to CD8^+^ T cells cultured alone (Figure 1l) [29]; this suggests a critical role for T_reg_-derived Gzm-B in killing of IL-2 effectors. Therefore, our data indicate that upregulation of cell-surface M6PR by IL-2 renders effector T cells vulnerable to T_reg_-derived Gzm-B lethal hit.

### M6PR downregulation by IL-7 renders effector T cells refractory to T_reg_-derived Gzm-B in the T_reg_-enriched tumor microenvironment *in vivo*

**Figure 2:**
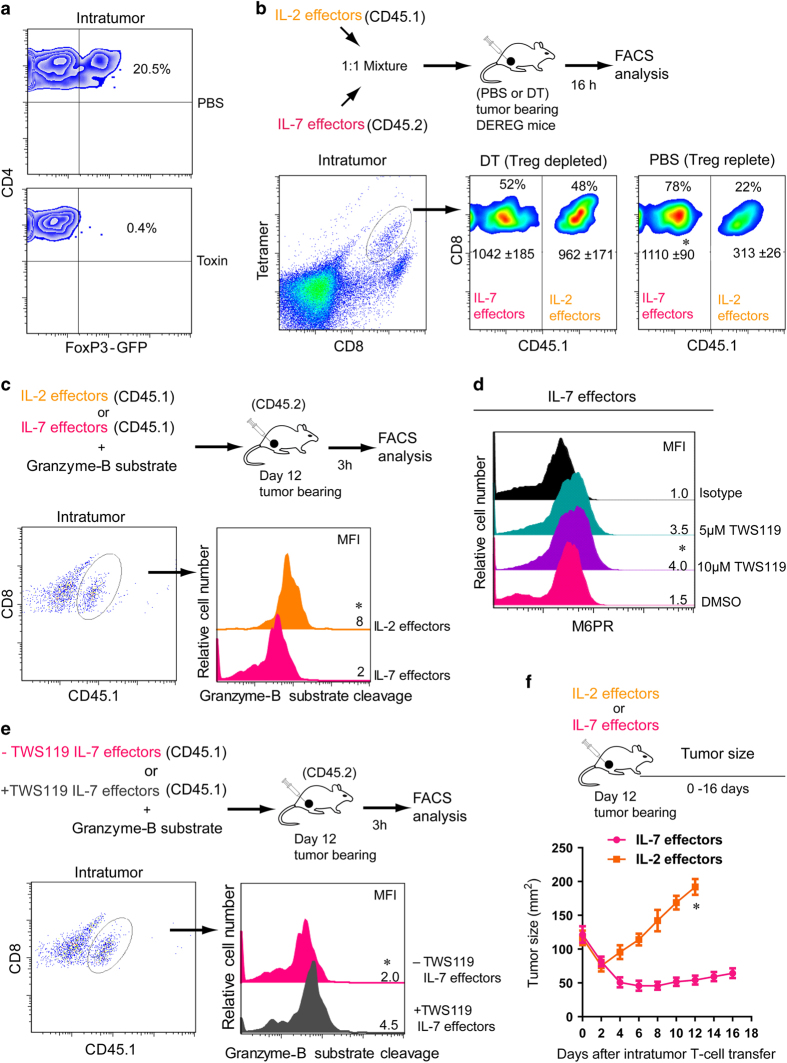
IL-7 effectors but not IL-2 effectors are refractory to T_reg_-derived Gzm-B lethal hit within the tumor microenvironment. (**a**) B16 melanoma (BL6-10 cells) subcutaneously injected into the thigh of transgenic DEREG (Foxp3-DTR) mice. After 12 days (tumors size, 100–130 mm^2^), DT (50 μg kg^−1^) was intraperitoneally administered at day −1 and day 0. Intratumoral T_reg_ cells (FoxP3^+^GFP expressing) were analyzed at day +1 using tumor cell suspension by flow cytometry. (**b**) The 1:1 mixture of IL-2(CD45.1) and IL-7(CD45.2) effectors were coinjected directly into the tumor of T_reg_-depleted (DT) or T_reg_-replete (phosphate-buffered saline-injected (PBS)) DEREG mice (*n*=6 per group). After 16 h, the ratio of recovered effector T cells was examined by gating on tetramer^+^CD8^+^ T cells. (**c**) IL-2(CD45.1) or IL-7(CD45.1) effectors with fluorogenic Gzm-B substrate were intratumorally injected into T_reg_-replete tumor-bearing mice (*n*=6). After 3 h, flow cytometry revealed Gzm-B activation in IL-2 effectors but not in IL-7 effectors. (**d**) Cell-surface M6PR expression of IL-7 effectors generated in the presence of indicated concentration of GSK-3β inhibitor (TWS119) or DMSO. (**e**) TWS119-treated or -untreated (CD45.1) IL-7 effectors plus Gzm-B substrate were intratumorally injected into T_reg_-replete tumor-bearing mice (*n*=6). TWS119-treated IL-7 effectors showed increased vulnerability to Gzm-B-mediated apoptosis. (**f**) OVA-specific tumor (melanoma, day 12) bearing mice (*n*=6 per group) intratumor injected with IL-2 or IL-7 effectors (4x10^6^ effectors per mouse). Tumor regression was followed over time. *P<0.05, versus cohorts of IL-7 effectors or TWS119-untreated IL-7 effectors. Data are representative of two-three independent experiments. MFI, mean fluorescence intensity.

T_reg_ cells preferentially accumulate within tumors [38], which provide an ideal T_reg_-enriched environment to investigate T_reg_ susceptibility of IL-2 and IL-7 effectors in an *in vivo* setting. Therefore, we generated T_reg_-replete tumor-bearing mice by subcutaneous thigh injection of OVA-expressing B16 melanoma (0.5×10^6^ cells per mouse) into DEREG (Foxp3-DTR) mice that express diphtheria toxin (DT) receptor-enhanced green fluorescent protein under control of the *Foxp3* promoter. Flow cytometry analysis of single-cell tumor samples revealed that ~20% of the intratumoral CD4^+^ T cells were FoxP3^+^GFP^+^ T_reg_ cells (Figure 2a). In such mice, a single dose of DT administration results in efficient ablation of T_reg_ cells [39]. In T_reg_-replete (phosphate-buffered saline-injected) or T_reg_-depleted (DT injected) tumor-bearing (day 12) DEREG mice, 1:1 mixture of congenic IL-2(CD45.1) and IL-7(CD45.2) effectors was intratumorally injected. After 16 h, mice were killed, and tumor cell-suspension samples were collected to investigate relative proportion of the two different subsets of effectors remaining within tumors. Flow cytometry demonstrated that intratumoral T_reg_ cells preferentially eliminated IL-2(CD45.1) effectors, whereas depletion of T_reg_ cells with DT prevented cell death of the effectors (Figure 2b). Taken together, our data indicate that IL-2 stimulation rather than lL-7 stimulation renders effector T cells vulnerable to T_reg_-mediated suppression *in vivo*.

To investigate Gzm-B-mediated killing of M6PR-expressing effector T cells within an *in vivo* T_reg_-enriched tumor environment, T_reg_-replete tumor-bearing mice were intratumorally injected with IL-2(CD45.1) or IL-7(CD45.1) effectors together with GranToxiLux, a cell-permeable fluorogenic substrate of Gzm-B. Mice were killed 3 h later, and tumors were collected for analysis. When Gzm-B enters the target cells, the substrate is cleaved, resulting in the emission of fluorescent light that represents Gzm-B-mediated cell apoptosis. Our flow cytometry analysis revealed more Gzm-B substrate cleavage in IL-2 effectors compared with IL-7 effectors (Figure 2c), indicating a preferential killing of M6PR^high^ IL-2 effectors by T_reg_ Gzm-B-mediated apoptosis and a preferential resistance of M6PR^low^ IL-7 effectors to T_reg_ Gzm-B-mediated killing within an *in vivo* T_reg_-enriched environment.

Glycogen synthase kinase-3β (GSK-3β) knockdown by siRNA has been shown to perturb anterograde transport of M6PR from trans-Golgi network to prelysosomal compartments, and to divert M6PR to the exocytic pathway, resulting in an increased cell-surface localization [40]. To further confirm that M6PR downregulation in IL-7 effectors shields them from T_reg_-mediated suppression, we treated IL-7 effectors with GSK-3β inhibitor to upregulate M6PR expression, and then assessed their Gzm-B susceptibility *in vivo*. Indeed, we found that pharmacological inhibition of GSK-3β by its inhibitor TWS119 resulted in the upregulation of cell-surface M6PR on IL-7 effectors (Figure 2d). IL-7(CD45.1) effectors cultured with or without TWS119 were then directly injected into tumors of tumor-bearing B6 (CD45.2) mice together with Gzm-B substrate GranToxiLux. Tumor samples were harvested 3 h after intratumoral injections for the analysis of IL-7 (CD45.1) effectors. Our flow cytometry analysis revealed an enhanced Gzm-B substrate cleavage in TWS119-treated mouse group (Figure 2e), indicating that cell-surface M6PR upregulation on TWS119-treated IL-7 effectors renders effectors susceptible to T_reg_ Gzm-B-mediated cell death, and thus confirming that cell-surface M6PR downregulation renders IL-7 effectors refractory to T_reg_ Gzm-B lethal hit. We next investigated whether the refractoriness of IL-7 effectors to T_reg_ cells will be advantageous in tumor therapy. We found that intratumoral transfer of IL-7 effectors but not IL-2 effectors caused a significant regression of tumor size in T_reg_-replete tumor-bearing mice (Figure 2f).

### IL-2 and IL-7 distinctly regulate M6PR transporter proteins KIF13A and β1-adaptin

**Figure 3:**
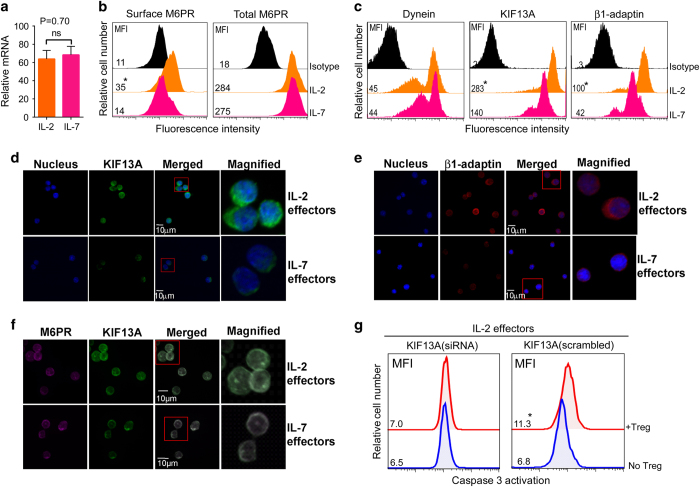
IL-2 and IL-7 differentially induce cell-surface M6PR expression by divergently regulating KIF13A-motorized transport machinery. (**a**) Relative M6PR mRNA in IL-2 and IL-7 effectors was quantified by real-time PCR. Glyceraldehyde 3-phosphate dehydrogenase (GADPH) was used as an endogenous control. (**b**) Cell-surface M6PR and total M6PR (surface+intracellular) were measured by flow cytometry. (**c**) Expression levels of dynein (important in retrograde transport of M6PR) and the motor proteins (KIF13A and β1-adaptin) involved in anterograde transport of M6PR were detected by intracellular staining. (**d**–**f**) Confocal images of IL-2 and IL-7 effectors revealed high expression of motor proteins KIF13A (**d**), β1-adaptin (**e**) as well as increased colocalization of KIF13A with M6PR (**f**) in IL-2 effectors. Cells were stained with nuclear stain (blue), KIF13A (green), β1-adaptin (red) and M6PR (magenta). Scale bars, 10 μm. (**g**) KIF13A knockdown (KIF13A siRNA lentivirus) or control (scrambled siRNA lentivirus) IL-2 effectors were cultured with or without T_reg_ cells (1:4) in the presence of cell-permeable fluorogenic caspase substrate (PhiPhiLux-G2D2) for 1 h, and caspase activation detected by flow cytometry. *P<0.05, versus cohorts of IL-7 effectors or effectors without T_reg_ cells. Data are representative of at least three independent experiments. MFI, mean fluorescence intensity.

We then investigated whether cell-surface M6PR expression is regulated transcriptionally or post-transcriptionally. Surprisingly, we found that M6PR expression in IL-2 and IL-7 effectors was very similar both at the mRNA (Figure 3a) and protein levels (Figure 3b, right panel). We were intrigued by this discrepancy, featuring similar total M6PR levels (Figure 3b, right panel) but dissimilar cell-surface M6PR expression (Figure 3b, left panel) [41]. The plus-end-directed microtubule motor-protein kinesin KIF13A transports M6PR budding off from the trans-Golgi network and then loading on the adaptor-protein β1-adaptin (a unit of the adaptor-protein-1 (AP-1) adaptor complex) onto the plasma membrane by microtubule-based transport machinery, whereas a dynein motor protein acts in retrograde M6PR transport [41]. We found that IL-2 and IL-7 induced a similar level of dynein expression (Figure 3c). Interestingly, intracellular staining (Figure 3c) and confocal imaging (Figures 3d and e) revealed not only a high expression of both KIF13A and β1-adaptin but also an enhanced colocalization of KIF13A with M6PR (Figure 3f) in IL-2 effectors but not IL-7 effectors. An overexpression of KIF13A has been reported to result in an increased localization of β1-adaptin and M6PR to the plasma membrane [41]. Thus, our data mechanistically reveal that the disparate cell-surface M6PR expression in IL-2 versus IL-7 effectors, despite their similar total M6PR levels, derives from a potentially enhanced KIF13A-motorized M6PR transport in the former compared with the latter.

Next, to investigate if high KIF13A expression predisposes IL-2 effectors to Gzm-B lethal hit, we knocked down KIF13A using pooled KIF13A siRNA lentivirus (piLenti-siRNA-GFP). IL-2 effectors transfected with KIF13A (scrambled) or KIF13A (siRNA) were cocultured with or without T_reg_ cells (1:4) for 1 h at 37 °C in the presence of PhiPhiLux-G2D2, a cell-permeable fluorogenic caspase-3 substrate (DEVDGI). Flow cytometry analysis revealed higher caspase-3 activation in KIF13A (scrambled)-transfected effectors but not in KIF13A (siRNA)-transfected effectors, confirming that enhanced KIF13A-motorized M6PR transport increases T-cell vulnerability to Gzm-B uptake-mediated cell apoptosis (Figure 3g).

### IL-2 but not IL-7 induces strong and prolonged activation of the PI3K-mTORC1 pathway

**Figure 4:**
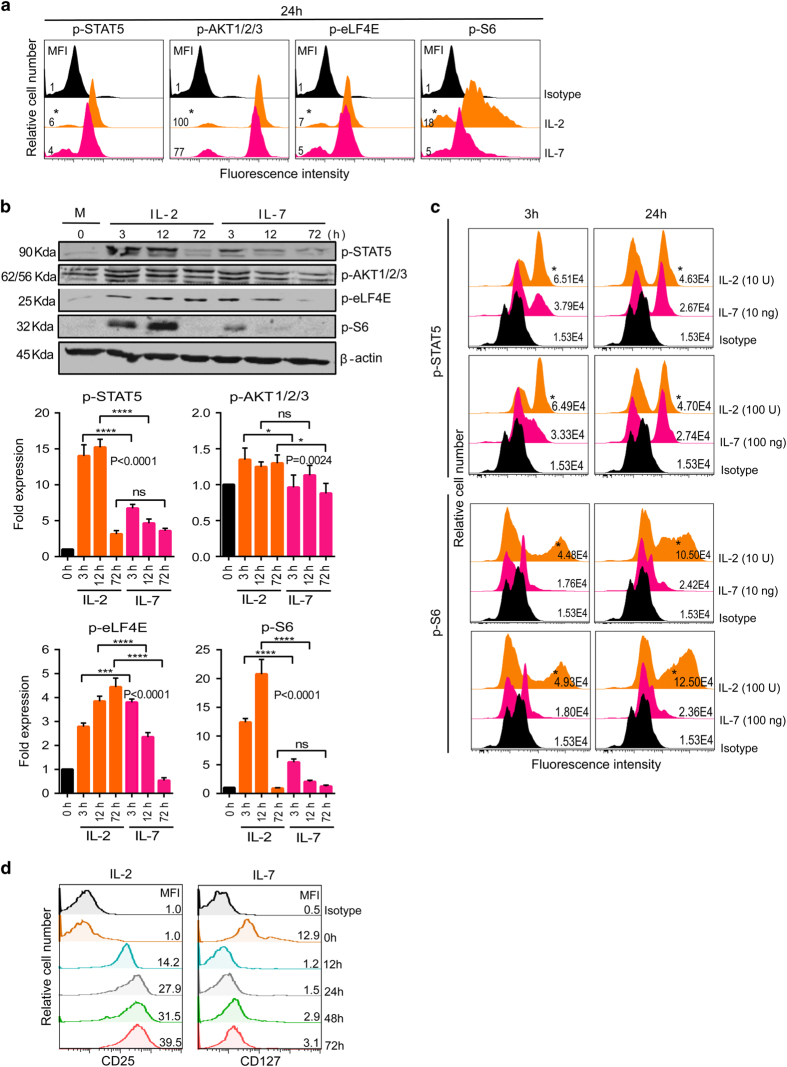
IL-2 rather than IL-7 induces strong and sustained signaling. (**a**) OT-I CD8^+^ T cells were activated with OVAI peptide (0.1 nM) in the presence of IL-2 (100 U ml^−1^) for 3 days, washed and rested overnight in cytokine-free medium, subsequently recultured in either IL-2 (100 U ml^−1^) or IL-7 (10 ng ml^−1^). After 24 h, the expression of pathway-specific effector molecules was detected by flow cytometry using intracellular staining. *P<0.05, versus cohorts of IL-7-stimulated T cells. (**b**) Cell lysates derived from recultured T cells for 3, 12 and 72 h were analyzed by western blots. The β-actin serves as a loading control. M, medium control without cytokine. Fold expression represents the ratio of expression of each molecule in cytokine-cultured T cells versus that in control medium-cultured T cells (0 h). (**c**) Comparison of low and high cytokine doses (10-fold range) to evaluate IL-2 and IL-7 induced signal strength. (**d**) IL-2 R or IL-7 R expression was measured by flow cytometry at indicated times in OT-I CD8^+^ T cells activated with OVAI peptide plus IL-2 (100 U ml^−1^) or IL-7 (10 ng ml^−1^). Results are mean±s.d. (error bars). Data are representative of three independent experiments. MFI, mean fluorescence intensity.

As γ_C_-family cytokines all signal through the phosphatidylinositol 3-kinase (PI3K)-mTOR and Janus kinase-signal transducer and activator of transcription (STAT) pathway [9, 31], we further compared the signal pathways in IL-2 and IL-7 effectors to explore the molecular basis for the divergent expression of KIF13A and AP-1, and thereby the differential cell-surface M6PR in CD8^+^ T cells. Our flow cytometry (Figure 4a) and western blot (Figure 4b) analyses revealed that IL-7 stimulated a relatively weak phosphorylation of Akt (a protein kinase-B downstream of PI3K), ribosomal protein S6 (S6, downstream of mTORC1, regulates protein translations) and eukaryotic translation initiation factor-4E (downstream of mTORC1, regulates initiation of protein translations). This is in contrast to IL-2 that activated enhanced STAT5 (downstream of Janus kinase), and induced heightened mTORC1 signaling (a positive regulator of cell proliferation but a negative regulator of CD8^+^ T-cell survival and memory) [42]. To rule out the possibility that differential pSTAT5 and pS6 expression in IL-2 and IL-7 effectors is due to cytokine dose-dependent signal strength, rather than an inherent difference between cytokines IL-2 and IL-7, we increased the concentration of IL-7 from 10 to 100 ng ml^−1^ and decreased the level of IL-2 from 100 to 10 U ml^−1^ so as to increase and decrease the signal strength in IL-7 and IL-2 effectors, respectively. Remarkably, flow cytometry data revealed that IL-7 effectors failed to upregulate pS6 expression significantly despite a 10-fold increase in the signal strength (Figure 4c). Similarly, even after decreasing a 10-fold signal strength, IL-2 effectors showed a significantly high pS6 expression both at 3 and 24 h (Figure 4c). These data refute the idea that IL-7 induced weak mTORC1 (pS6) is due to low-dose signal strength. It is worth mentioning that the differential signal strength of IL-2 and IL-7 was also found related to the expression pattern of their unique receptors. In agreement with previous findings, we found that IL-2 enhanced its receptor (IL-2Rα) (Figure 4d, left panel) that enabled its own signal amplification, whereas IL-7 signaling downregulated IL-7Rα (Figure 4d, right panel), thereby decreasing its signal strength [25]. Consistent with previous studies [42], our data suggest that though both IL-2 and IL-7 initiates similar signaling cascades [8, 25], they are intrinsically different in controlling the signal strength. IL-2 amplifies its signal strength by positively regulating its own receptor, whereas IL-7 weakens its signal strength by downregulating its own receptor.

### PI3K-mTORC1 pathway critically regulates KIF13A and M6PR surface transport

**Figure 5:**
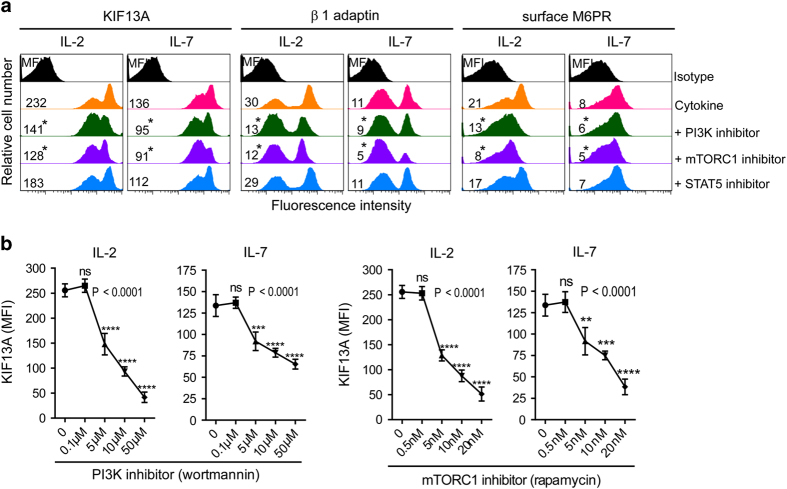
PI3K/mTORC1 signal pathway critically regulates KIF13A and its transport machinery. (**a**) OT-I CD8^+^ T cells were activated with OVAI peptide (0.1 nM) in the presence of IL-2 (100 U ml^−1^) for 3 days, then rested overnight in cytokine-free media, and subsequently treated with or without pathway-specific inhibitors (5 μm wortmanin, a PI3K inhibitor; 5 nM rapamycin, mTORC1 inhibitor; 50 μm STAT5 inhibitor), for at least 1 h before cytokine stimulation. Intracellular KIF13A and β1-adaptin and surface M6PR were analyzed after 24 h by flow cytometry. *P<0.05, versus cohorts of cytokine-stimulated T cells without inhibitor treatment. (**b**) Wortmanin and rapamycin inhibited KIF13A expression in a dose-dependent manner. Results are mean±s.d. (error bars). Data are representative of at least three independent experiments. MFI, mean fluorescence intensity.

We next sought to identify the molecular pathway that has a critical role in KIF13A-motorized M6PR expression by using pathway-specific inhibitors. Our flow cytometry data demonstrated that the inhibition of STAT5 (by STAT5 inhibitor) did not significantly affect either KIF13A or β1-adaptin expression. Interestingly, pharmacological inhibition of PI3K (by inhibitor wortmannin) or its downstream effector mTORC1 (by inhibitor rapamycin) significantly suppressed the expression of KIF13A and β1-adaptin, and thereby cell-surface M6PR expression, in both IL-2 and IL-7 effectors (Figure 5a). We found that P13K and mTORC1 inhibitors downregulated KIF13A expression in a dose-dependent manner (Figure 5b), which is in agreement with previous studies showing an important role for PI3K signaling in cell-surface M6PR expression [43] and KIF13A-mediated abscission during mitosis [31]. Thus, our results reveal that P13K-mTORC1 pathway has a major role in regulating the expression of KIF13A and β1-adaptin, and thereby M6PR cell-surface transport in T cells.

### Rapamycin treatment regulates KIF13A-motorized M6PR and T-cell fates

**Figure 6:**
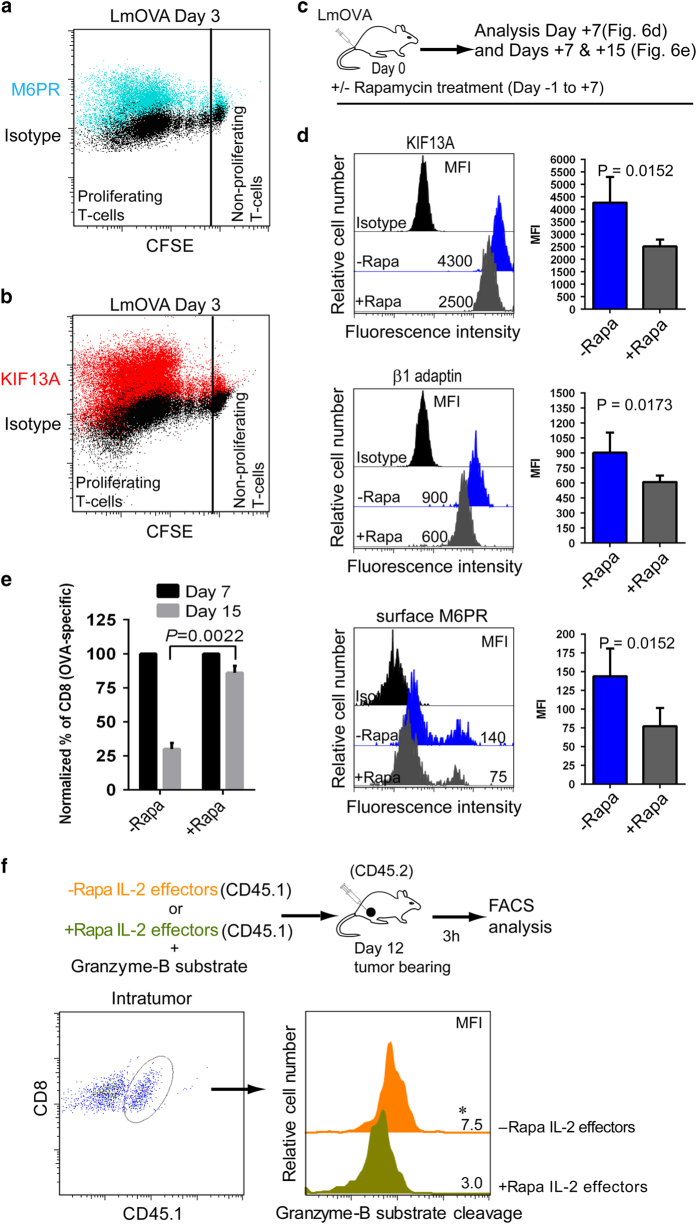
Rapamycin-mediated downregulation of KIF13A-motorized M6PR leads to reduced T-cell contraction and decreased T-cell vulnerability to T_reg_-cell-derived Gzm-B in tumor. (**a**) Cell-surface M6PR and (**b**) intracellular KIF13A expression in CFSE (4 μM)-labeled OT-I cells (4×10^6^ per mouse) recovered from the spleen (*n*=4) after 3 days of LmOVA (2500 CFU per mouse) challenge (M6PR (cyan) or KIF13A (red) overlaid on isotype staining (black)). (**c**) B6 mice were treated (+Rapa, *n*=6 mice) or not treated (−Rapa, *n*=6 mice) with mTORC1 inhibitor, rapamycin (75 μg kg^−1^ body weight), during LmOVA challenge (days −1 to+7), (**d**) KIF13A, β1-adaptin and surface M6PR expression were analyzed in CD8^+^tetramer^+^ T cells (blood) and (**e**) Frequency of CD8^+^tetramer^+^ T cells remaining on day 15 relative to that on day 7, set as 100%. (**f**) IL-2(CD45.1) effectors generated in the presence or absence rapamycin were injected directly into the tumor (day 12) along with a cell-permeable fluorogenic Gzm-B substrate. After 3 h, tumor cell suspensions were analyzed by flow cytometry by gating on OVA-specific CD45.1^+^CD8^+^ T cells to detect Gzm-B substrate cleavage. *P<0.05, versus cohorts of rapamycin-treated IL-2 effectors. Results are mean±s.d. (error bars). Data are representative of three independent experiments. MFI, mean fluorescence intensity.

We wanted to investigate the possibility of regulating KIF13A and KIF13A-motorized M6PR under a physiological condition using a mouse model of *L. monocytogenes* infection. Indeed, we found that during the expansion phase of immune response, proliferating CD8^+^ T cells upregulated not only the cell-surface M6PR (Figure 6a) as we have seen before [3] but also the intracellular KIF13A (Figure 6b). Elevated KIF13A expression is not very surprising, as a recent study reported that KIF13A helps cellular proliferation by regulating cytokinesis [31], and thus KIF13A should be elevated in activated T cells to facilitate cellular proliferation. This result also explains our previous report that shows almost all proliferating T cells express high cell-surface M6PR [3], as M6PR depends on KIF13A for its cell-surface transport [41]. Moreover, we also observed that T cells with enhanced IL-2 receptor (CD25) expressed significantly more KIF13A (Supplementary Figure S3), probably because of high mTORC1 activity through IL-2 signaling.

We next examined the possibility of drug-mediated regulation of KIF13A and KIF13A-motorized M6PR during infection. Our *in vitro* experiments clearly showed that the P13K-mTORC1 pathway has a critical role in regulating M6PR transporters, KIF13A and β1-adaptin. To target mTORC1 kinase, we used rapamycin (a drug) to inhibit potently the mTORC1 activity [14]. Mice were injected with rapamycin during *L. monocytogenes* infection (−1 to +7 days) (Figure 6c), as described previously [14]. Indeed, we found a significant downregulation of KIF13A, β1-adaptin and cell-surface M6PR in OVA-specific CD8^+^ T cells on day 7 of infection, when Gzm-B level remains very high (Figure 6d) [9], as well as a significantly reduced T-cell contraction (day 15) (Figure 6e). Altogether, these results suggest that pharmacological inhibition of mTORC1 can control KIF13A and KIF13A-motorized M6PR, thereby M6PR regulated T-cell fates *in vivo*.

To gain greater insights into the role of mTORC1 inhibition in T-cell effectors in determining Gzm-B-mediated T-cell fates *in vivo*, we further designed an experiment in tumor-bearing mice, and tested Gzm-B susceptibility of adoptive IL-2 effectors (with or without mTORC1 inhibition) within tumors with enriched immunosuppressive T_reg_ cells. T_reg_-replete tumor-bearing mice were intratumorally injected with rapamycin-treated or non-treated IL-2 (CD45.1) effectors together with GranToxiLux, and 3 h later, mice were killed to collect tumors for analysis (Figure 6f). Flow cytometry revealed reduced Gzm-B substrate cleavage in mTORC1-inhibited (rapamycin-treated) IL-2 effectors (Figure 6f), confirming a critical role for mTORC1 in regulating Gzm-B susceptibility of T-cell effectors within the tumor microenvironment. These data thus strongly support our notion that T-cell fate control can be achieved by regulating the mTORC1-KIF13A-M6PR axis *in vivo*.

## Discussion

Identifying the key factors that dictate T-cell fates is crucial for manipulating immune responses. It was proposed, more than a decade ago, that M6PR can have role in T-cell immune function [15, 44]. Despite great progress in T-cell biology, the function and regulation of M6PR in T cells remain poorly understood. Emerging evidence in the field associating M6PR to immune regulation [3, 22, 45] and also to immune dysregulation [24, 46] has led to renewed interests in exploring the role of M6PR in the immune system.

It is now well recognized that T cells integrate numerous signals temporally and spatially during immune responses [6], wherein cell-extrinsic factors, like cytokines, have critical roles in shaping these responses [8, 25, 47–49]. In particular, the two best-studied γ_c_-family cytokines IL-2 and IL-7 are important in the process of T-cell survival and memory generation [28, 47], but opposing effects of IL-2 and IL-7 in T-cell susceptibility to T_reg_-mediated suppression are also documented [29, 30]. We recently found that T_reg_ cells suppressed M6PR^high^ effectors but not of M6PR^low^ effectors via T_reg_ Gzm-B-induced cell apoptosis in *L. monocytogenes*-infected mice [3], leading to an assumption that cytokine-mediated divergent T-cell fates may be dictated via M6PR regulation.

In this study, we assessed cell-surface M6PR expression on IL-2 and IL-7 effectors. In support of our assumption, we first show that indeed IL-2 and IL-7 stimulate M6PR^high^ and M6PR^low^ effectors, respectively. We next assessed the vulnerability of IL-2 and IL-7 effectors to T_reg_-mediated cell death by using an *in vitro* coculture system, in which 1:1 mixture of M6PR^high^ IL-2 and M6PR^low^ IL-7 effectors was simultaneously cocultured with T_reg_ cells. We clearly demonstrate that T_reg_ cells preferentially kill M6PR^high^ IL-2 effectors, whereas M6PR^low^ IL-7 effectors are refractory to T_reg_ suppression, suggesting that an upregulation of cell-surface M6PR on effectors increases their vulnerability T_reg_ Gzm-B lethal hit. This is further confirmed by our finding of a decreased susceptibility of IL-2 effectors with siRNA-mediated knockdown of M6PR.

Using a subcutaneous tumor model (B16 melanoma), we next provided an *in vivo* evidence for divergent T-cell fates after adoptive transfer of effectors into tumors with enriched immunosuppressive T_reg_ cells of tumor-bearing mice. We demonstrate that tumor-resident T_reg_ cells preferentially eliminate M6PR^high^ IL-2 effectors rather than M6PR^low^ IL-7-effectors through T_reg_ Gzm-B-mediated cell apoptosis. GSK-3β inhibitor-mediated upregulation of M6PR on IL-7 effectors renders them vulnerable to T_reg_ Gzm-B-mediated lethal hit. These data are in agreement with previous studies showing a critical role for M6PR in Gzm-B-mediated cell death [3, 15, 17, 46]. We found that M6PR^low^ IL-7 effectors are shielded against T_reg_ suppression and thereby able to regress the tumor size on adoptive T-cell transfer substantially. In contrast, M6PR^high^ IL-2 effectors are preferentially killed by T_reg_ Gzm-B-mediated lethal hit and failed to control tumor growth within the immunosuppressive tumor microenvironment. These data suggest that modulation of M6PR recruitment onto T-cell surfaces by cytokine or any other suitable means could be a novel mechanism for regulating T-cell fates.

We next elucidated a potential mechanism by which IL-2 and IL-7 differentially regulate M6PR expression on T cells. Interestingly, we show that enhanced cell-surface M6PR expression on IL-2 effectors is not a result of increased M6PR protein synthesis, but a consequence of overexpression of kinesin motor-protein, KIF13A and adaptor-protein β1-adaptin, which facilitate anterograde transport of M6PR onto the plasma membrane. This is supported by our experiments using siRNA-mediated KIF13A knockdown that show a decreased vulnerability of IL-2 effectors to T_reg_ Gzm-B lethal hit. These results are in agreement with a previous study showing that an overexpression of KIF13A results in an increased localization of β1-adaptin and M6PR to the plasma membrane [41].

We further sought to identify the molecular pathway that has a critical role in KIF13A-motorized M6PR expression. Using signal pathway-specific inhibitors, we demonstrate that a strong and prolonged PI3K/mTORC1 signaling activated by inflammatory cytokine IL-2 leads to an upregulation of KIF13A/β1-adaptin and cell-surface M6PR, rendering the M6PR^high^ IL-2 effectors vulnerable to T_reg_ Gzm-B-mediated cell death, whereas a weak PI3K/mTORC1 signaling activated by prosurvival cytokine IL-7 fails to upregulate KIF13A and β1-adaptin, the crucial machinery of M6PR transport, leading to M6PR^low^ IL-7 effectors resistant to T_reg_ Gzm-B-mediated cell death. Using 10-fold higher or lower doses of cytokines, we demonstrated that the divergent regulation of cognate receptors but not the cytokine doses dictates differential signal strengths of IL-2 vis-à-vis IL-7. Hence, in antigen-activated T cells, temporal and quantitative differences between IL-2- and IL-7-induced PI3K/mTORC1 signaling pathways result in significant difference in KIF13A-mediated M6PR transport.

Having shown *in vitro* that mTORC1 kinase upregulates KIF13A, we next examined the possibility of regulating KIF13A and KIF13A-motorized M6PR in a physiological condition. A recent study reported that KIF13A, which transports M6PR onto the plasma membrane [41], helps cellular proliferation by regulating cytokinesis [31]. Thus, we hypothesized that KIF13A should be elevated in activated T cells to facilitate cellular proliferation. Indeed, we show that rapidly proliferating T cells significantly upregulate KIF13A in *L. monocytogenes*-infected mice. In agreement with our previous study [3], we also demonstrate concurrent upregulation of M6PR on T cells after the infection.

Using the *L. monocytogenes* bacterial challenge model (LmOVA), we further show that *in vivo* rapamycin-mediated inhibition of mTORC1 results in the suppression of KIF13A and β1-adaptin and KIF13A-motorized M6PR on effector T cells, and *in vivo* rapamycin treatment also significantly augments the survival of effector T cells during the contraction phase. Notably, using tumor mouse model, we provide a definite evidence that mTORC1 inhibition by rapamycin shields T cells against T_reg_-derived Gzm-B lethal hit within the T_reg_-enriched tumor microenvironment. These findings are consistent with our recent study in mice showing that M6PR^low^ effectors rather than M6PR^high^ effectors survive better *in vivo* and provide protection against intravenous tumor challenge [3]. Thus, these data may suggest that mTORC1 inhibition can improve survivability of effector T cells *in vivo*, at least in part through decreasing T-cell vulnerability to Gzm-B lethal hit. In addition to facilitating Gzm-B-mediated cell apoptosis [15, 17, 46], M6PR also activates inhibitory cytokine-transforming growth factor-β and degrades insulin-like growth factor-II, depriving cells of local growth mitogens [15]. Overall, our finding suggests that M6PR downregulation by mTORC1 inhibition may improve T-cell quality by shielding T cells from several inhibitory networks.

The differential stimulatory strength in immune responses is associated with T-cell fate and memory: strong stimuli induce generation of short-lived effectors and moderate stimuli promote long-term memory T-cell generation [50, 51]. This view was further supported by the findings that (i) the distinct strength of antigen stimulation [52, 53] or activation of the AKT-mTORC1 pathway [14, 54, 55], (ii) the different length of IL-2 signaling [26, 27] and (iii) the different cytokine (IL-2 vs IL-7) signaling [28] control the formation of short-life effector and long-term memory T cells. However, their underlying molecular mechanisms for the intrinsic link between the above observations are still elusive. Our previous study unequivocally demonstrated that M6PR^low^ effectors preferably survive the early phase of contraction to seed the initial memory T-cell pool [3], when *in vivo* Gzm-B level remains very high [14]. In this study, we demonstrate that inflammatory cytokine IL-2 activates a strong mTORC1 signaling, leading to an upregulation of KIF13A and KIF13A-motorized M6PR on T-cell surfaces and formation of short-lived effectors. Our data thus provide some mechanistic insights into recent studies reporting that CD8^+^ T cells receiving a strong IL-2 signaling (due to prolonged IL-2Rα expression) generate short-lived effectors and are eliminated early during the contraction phase, whereas those receiving a weak IL-2 signaling (due to transient IL-2Rα expression) survive longer [26, 27]. In agreement with this concept, here we determine that IL-7-induced weak mTORC1 or inhibition of mTORC1 by rapamycin generates M6PR^low^ effectors with improved ability to survive within the immunosuppressive microenvironment. These findings corroborate the concept that the differential strength of AKT/mTOR signaling regulates effector and memory differentiation, wherein sustained activation of AKT or inhibition of mTORC1 drives differentiation of terminal effectors or memory T cells, respectively [14, 54, 55]. Our study thus offers novel insights into understanding the critical role for these cytokines in the regulation of T-cell fates.

**Figure 7:**
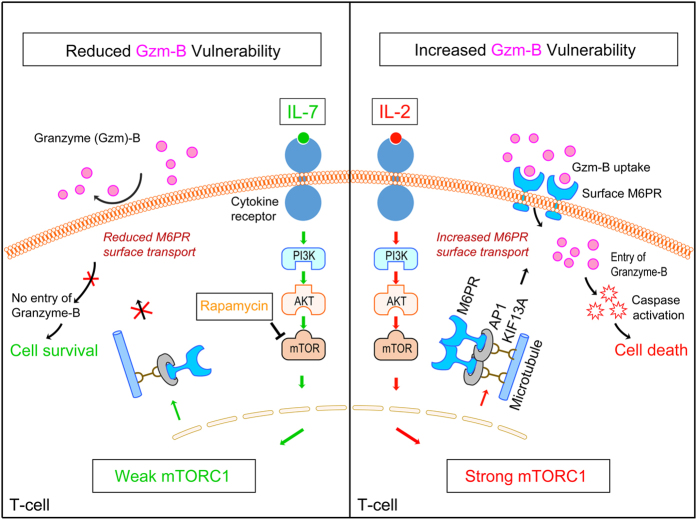
Schematic diagram of M6PR-mediated regulation of T-cell fates via mTORC1-KIF13A-M6PR axis. Strong mTORC1, as induced by pro-inflammatory cytokine IL-2, enhances KIF13A and β1-adaptin (AP-1)-mediated transport of M6PR onto the cell-surface, rendering T-cell vulnerable to Gzm-B uptake-mediated cell-death (right). In contrast, mTORC1 inhibition by rapamycin (a drug) or weak mTORC1 as induced by IL-7, prevents KIF13A and β1-adaptin-mediated transport of M6PR onto the cell-surface, making T-cell refractory to Gzm-B mediated cell-death (left).

Taken together, our findings demonstrate that distinct regulation of the PI3K-mTORC1-KIF13A axis is a key mechanism underlying the phenomenon of differential expression of cell-surface M6PR, which is critical in determining T-cell resistance or susceptibility to T_reg_-mediated suppression (Figure 7). A deeper understanding of factors and mechanisms that regulate the PI3K-mTORC1-KIF13A axis, a potential target for the immune regulation, is warranted to manipulate optimally T_reg_-mediated suppression in clinical settings.

## Materials and methods

### Study design

Mice were purchased from the Jackson Laboratory, Bar Harbor, ME, USA and maintained at Health Sciences animal facilities, University of Saskatchewan under specific pathogen-free conditions. All experiments were performed according to protocols and guidelines approved by the Animal Research Ethics Board, University of Saskatchewan. All experiments included four to six mice per group and were repeated at least two to three times.

### Mice, antibodies and reagents

WT C57BL/6, CD45.1^+^ (B6.SJL-Ptprc^a^Pepc^b^/BoyJ), OVA peptide (OVA_257–264_)-specific TCR transgenic OT-I (C57BL/6-Tg (TcraTcrb)1100Mjb/J) (OT-I/CD45.2^+^) mice and transgenic DEREG mice that express a simian DT receptor-enhanced green fluorescent protein fusion protein under control of the *Foxp3* promoter were purchased from the Jackson Laboratory. OT-I/CD45.1^+^ mice were derived from breeding OT-I (C57BL/6-Tg (TcraTcrb) 1100Mjb/J) with CD45.1^+^ (B6.SJL-Ptprc^a^Pepc^b^/BoyJ) mice. Antibodies include anti-M6PR (R&D Systems, Minneapolis, MN, USA), anti-Ki-67 (Abcam, Toronto, ON, Canada), anti-KIF13A, anti-β-adaptin, anti-p-AKT1/2/3 (Ser473) and anti-β-actin (Santa Cruz, Dallas, TX, USA), anti-dynein (Novus Biologicals, Oakville, ON, Canada), anti-p-STAT5 (Tyr694), anti-p-eukaryotic translation initiation factor-4E (Ser209) and anti-p-S6 (Ser235/236) (Cell Signalling, Danvers, MA, USA), anti-CD8, anti-CD45.1, anti-CD45.2, anti-FoxP3, anti-CD25, anti-CD127, anti-perforin, anti-CXCR3, anti-CD62L and anti-CD44 (eBioscience, San Diego, CA, USA), anti-Bcl-2, anti-IL-2 and anti-Gzm-B (BD Bioscience). Inhibitors include STAT5 inhibitor (Calibiochem, San Diego, CA, USA), wortmannin and GSK-3β Inhibitor XII, TWS119 (Millipore, Billerica, MA, USA) and rapamycin (Sigma, Markham, OT, Canada ). Other reagents include Gzm-B substrate GranToxiLux (OncoImmunin, Gaithersburg, MD, USA), CFSE and ProLong Diamond AntifadeMountant with 4',6-diamidino-2-phenylindole (Molecular Probes, Eugene, OR, USA) and cytokines IL-7 (eBioscience) and IL-2 (PeproTech, Embrun, ON, Canada).

### T_reg_-cell preparation

CD4^+^CD25^+^ T_reg_-cells were purified from splenocytes of WT C57BL/6 mice using CD4^+^CD25^+^ regulatory T-cell Isolation Kit (Miltenyi Biotech) and following manufacturer’s directions, and followed by stimulation with anti-CD3/CD28 antibody-coated latex beads (MACS, Auburn, CA, USA) plus IL-2 (100 U ml^−1^) for 3 days [29]. Subsequently, T_reg_-cells were analyzed for FoxP3, Gzm-B and perforin expression and then used in T-cell coculture and adoptive T-cell transfer assays [29].

### IL-2- and IL-7-effector preparation

CD8^+^ T cells were purified (>95% purity) from splenocytes of major histocompatibility complex class I-restricted OVA-specific TCR transgenic OT-I (CD45.2^+^) or OT-I/CD45.1^+^ mice by using CD8^+^ T-Cell Isolation Kit (StemCell, Vancouver, BC, Canada). Naïve CD8^+^ T cells were then stimulated *in vitro* with OVAI peptide (OVA_257–264_, SIINFEKL) (0.1 nM) plus IL-2 (100 U ml^−1^) for 3 days, followed by another 2 days of culturing in IL-2 (100 U ml^−1^) and IL-7 (10 ng ml^−1^) [56], forming IL-2 and IL-7 effectors, respectively. IL-2 and IL-7 effectors were analyzed for cell-surface markers CD44, CD62L, CD25, CD127 and CXCR3, as well as intracellular Bcl-2 and IL-2 expression by flow cytometry. Samples for IL-2 expression analysis were treated with BD GolgiStop (protein transport inhibitor) as per BD protocol during the last 10 h of culturing. In other experiments, IL-2 effectors were cultured in the presence of rapamycin to generate M6PR downregulated IL-2 effectors. In some experiments, T-cell effectors were cultured in the presence of GSK-3β inhibitor, TWS119 (5 or 10 μM), to generate IL-7 effectors with M6PR^high^ phenotype. In some experiments, OT-1 CD8^+^ cells were stimulated with 10 U ml^−1^ IL-2 or 100 ng ml^−1^ IL-7 for 3 and 24 h.

### M6PR and KIF13A knockdowns in CD8^+^ T cells using lentivirus-based siRNA

Spleen-purified naive OT-I CD8^+^ T cells were stimulated *in vitro* with OVAI peptide (OVA_257–264_, SIINFEKL) (0.1 nM) plus IL-2 (100 U ml^−1^) for 24 h. Then, IL-2 effectors were transduced by lentiviral particles (piLenti-siRNA-GFP from ABM Inc., Richmond, BC, Canada). Pooled KIF13A siRNA lentiviruses and pooled M6PR siRNA lentiviruses were used to knockdown KIF13A and M6PR, respectively, in IL-2 effectors. Scrambled siRNA lentiviruses were used as control. Lentiviral transduction was carried out using multiplicity of infection of 10 in the presence of 10 μg ml^−1^ polybrene with the plate spun in a Beckman Allegra 6R centrifuge (Beckman Coulter, Mississauga, ON, Canada) at 1 200 r.p.m. for 1 h at 30 °C. After removing supernatant, growth medium containing OVAI peptide (OVA_257–264_, SIINFEKL) (0.1 nM) plus IL-2 (100 U ml^−1^) was added to the well. The knockdown efficiency was confirmed by quantitative real-time PCR, GFP expression and western blot. Knockdown or control IL-2 effectors were used for experiments after 72 h.

### Western blot and flow cytometry analysis of signaling molecules

Purified naive OT-I CD8^+^ T cells were stimulated *in vitro* with OVAI peptide (OVA_257–264_, SIINFEKL) (0.1 nM) plus IL-2 (100 U ml^−1^) for 3 days, washed and rested overnight in cytokine-free medium, followed by restimulation with either IL-7 (10 ng ml^−1^) or IL-2 (100 U ml^−1^) for 0, 3, 12, 24 or 72 h. T-cell samples harvested after 24 h stimulation were analyzed by flow cytometry, and the other samples analyzed by western blotting, as indicated in the figure legends, and as shown in the figures. Some other experiments, OVA1-peptide-stimulated OT-I CD8^+^ T cells (3 days culture in IL-2 (100 U ml^−1^) were washed and rested overnight in cytokine-free medium, followed by restimulation with either IL-7 (10 or 100 ng ml^−1^) or IL-2 (10 or 100 U ml^−1^) for 0, 3 and 24 h and intracellular pS6 and pSTAT5 detected by flow cytometry. In the study with signal pathway-specific inhibitors, T-cell samples treated with signal-specific inhibitors such as STAT5 inhibitor (50 μM), wortmannin for PI3K (0.1, 5, 10 and 50 μM) and rapamycin for mTORC1 (0.5, 5, 10 and 20 nM) for at least 1 h before restimulation with either IL-7 (10 ng ml^−1^) or IL-2 (100 U ml^−1^) for 24 h were also analyzed by western blotting. In western blotting, CD8^+^ T-cell lysates were prepared using whole-cell extraction protocol containing phosphatase and protease inhibitors (Active Motif, Carlsbad, CA, USA). Standard procedures were followed for sodium dodecyl sulfate gel electrophoresis, nylon membrane transfer, immunoblotting and development of blots by LI-COR Odyssey Scanner (LI-COR, Lincoln, NE, USA).

### Analysis of IL-2 and IL-7 effectors after T-cell coculture *in vitro*

Congenic IL-2 (CD45.1) and IL-7 (CD45.2) effectors at 1:1 ratio were cocultured in the presence or absence of T_reg_ cells (effectors:T_reg_, 1:4) for overnight at 37 °C. T cells were collected for flow cytometry analysis to examine the ratio of recovered IL-2 and IL-7 effectors. In other experiment to examine T_reg_-mediated apoptosis of effector T cells, 1:1 mixture of IL-2(CD45.1) and IL-7(CD45.2) effectors was cocultured with or without T_reg_ cells (effectors:T_reg_, 1:4) for 60 min at 37 °C in the presence of PhiPhiLux-G2D2, a cell-permeable fluorogenic caspase-3 substrate (DEVDGI), following the kit (OncoImmune) protocol. After incubation with T_reg_ cells, recovered cells were analyzed using flow cytometry that detects the fluorescent light emitted due to the cleavage of fluorogenic caspase-3 substrate in the target cells undergoing apoptosis. In experiments determining the role of M6PR or KIF13A in T_reg_-cell-mediated apoptosis of IL-2 effectors, M6PR knockdown or KIF13A knockdown or Scrambled (control) IL-2 effectors were cocultured with T_reg_ cells (1:4) for 60 min at 37 °C in the presence of PhiPhiLux-G2D2. Flow cytometry analysis was carried out to detect caspase-3 activation in IL-2 effectors as an indicator of apoptosis.

### Analysis of IL-2 and IL-7 effectors after adoptive T-cell transfer into mouse tumor model

Mouse B16 melanoma BL6-10 cells (0.5×10^6^ cells per mouse) were subcutaneously injected into the right thigh of mice. Twelve days after the subcutaneous tumor challenge, tumor size was about 100–130 mm^2^. To assess T_reg_-mediated killing of effectors *in vivo*, T_reg_-replete or -deplete mice (*n*=6 per group; 2 mice per trial) bearing tumors (100–130 mm^2^) were intratumorally injected with 1:1 mixture of congenic IL-2(CD45.1) and IL-7(CD45.2) effectors. At 16 h after injection, C57BL/6 mice were killed and tumor collected for flow cytometry analysis. In experiments to examine Gzm-B-mediated killing of effectors, T_reg_-replete C57BL/6 mice (CD45.2) bearing tumors were intratumorally injected with CD45.1 congenic IL-2 or IL-7 effectors together with GranToxiLux, a cell-permeable fluorogenic substrate of Gzm-B, and following 3 h after injection, mice were killed, and tumors were collected for flow cytometry analysis. Gzm-B-mediated cell apoptosis was detected using flow cytometry, as Gzm-B substrate is cleaved and fluorescent light emitted. To assess the role of M6PR downregulation in the refractoriness of IL-7 effectors to Gzm-B-mediated cell death, IL-7 effectors (CD45.1) treated with GSK-3β inhibitor, TWS119 (5 μM), or left untreated, were injected intratumorally together with GranToxiLux into C57BL/6 (CD45.2) mice; mice were killed 3 h after injection, and tumors were collected for analysis. Next, to assess the role for mTORC1-regulated Gzm-B-mediated death of IL-2 effectors, rapamycin-treated (mTORC1-inhibited) or -untreated CD45.1 congenic IL-2 effectors were intratumorally injected together with GranToxiLux into C57BL/6 mice; mice were killed 3 h after injection, and tumors were collected for analysis. In DEREG (FoxP3-DTR) transgenic mice, T_reg_ cells were depleted as reported elsewhere [14] by intraperitoneal injection of DT (Sigma-Aldrich, St. Louis, MO, USA) (40 μg kg^−1^ body weight) (phosphate-buffered saline-injected mice served as control) on days −2 and −1 before the intratumoral injection of effector T cells.

### Intratumor T-cell transfer and tumor growth

Mice were injected with B16 melanoma (0.5×10^6^ cells per mouse subcutaneously) on right thigh. After 12 days, when tumor size were ~100–130 mm^2^, three recipient mice per trial per group (*n*=6 per group) were injected with IL-2 or IL-7 effectors (4×10^6^ cells per mouse) directly into tumors. Tumor size were recorded before T-cell adoptive transfer and every 2 days until day 16 or tumor reached 200 mm^2^ (mice were killed).

### Rapamycin treatment in mice challenged with LmOVA

LmOVA challenge (2500 colony-forming unit) was performed with published strain by intravenous injection in the tail vein [1, 3]. C57BL/6 mice were administered daily with rapamycin (Sigma) (75 μg kg^−1^ body weight) during the T-cell expansion phase (day −1 before LmOVA infection to day +7 postinfection) [14]. On days 7 and 15 after LmOVA infection, mouse tail blood samples were stained with PE-tetramer and FITC-anti-CD8 antibody, and analyzed for assessment of OVA-specific CTL responses by flow cytometry. Intracellular expression of KIF13A and AP-1 was detected using the kit from BD Bioscience, and surface M6PR expression in OVA-specific CTL was detected on day 7 post-LmOVA infection.

### Flow cytometry

Flow cytometry data were acquired by FACSCaliber (BD Bioscience), EpicsXL (Beckman Coulter, Ashland, OR, USA), MoFlo XDP (Beckman Coulter), CytoFlEX (Beckman Coulter) and analyzed with the FlowJo software (TreeStar, Ashland, OR, USA). Intracellular staining was carried out as per the manufacturer's (BD Bioscience) protocol by flow cytometry [3].

### Confocal microscopy

IL-2 or IL-7 effectors were cytospun onto slides. Cells were fixed, permeabilized (Cytofix/Cytoperm Kit, BD Bioscience, , San Jose, CA, USA) and stained with primary antibodies 1:200 diluted in permeabilization buffer for 30 min at room temperature, washed three times, stained with secondary antibodies (Alexa Fluor-488 or R-PE conjugated) for 30 min at room temperature, washed three times, and then preserved in ProLong Diamond Antifade Mountant with 4',6-diamidino-2-phenylindole (Molecular Probes). Confocal images of fixed slides were taken using the Zeiss LSM700 confocal microscope (Carl Zeiss, Oberkochen, BW, Germany) with ×20 objective.

### Real-time PCR analysis of M6PR expression

Total RNA was isolated from 2×10^6^ cells (IL-2 or IL-7 effectors) after 12 h of cytokine stimulation. cDNA was prepared using RT-PCR Kit (Qiagen, Germantown, MD, USA). Using SYBR Green Master Mix (Qiagen), and primers for M6PR (MQP029633) and glyceraldehyde 3-phosphate dehydrogenase (MQP027158) (Genecopoeia, Rockville, MD, USA), real-time PCR analysis was carried out as per the manufacturers’ protocol. Relative mRNA was calculated by ΔCt method using glyceraldehyde 3-phosphate dehydrogenase as the control.

### Statistical analysis

Statistical analyses were performed using the unpaired *t*-test (two-tailed) or analysis of variance for comparison of means using the GraphPad Prism6 software (GraphPad, La Jolla, CA, USA). Values of *P*<0.05 and *P*<0.01 were considered to be statistically significant and very significant.
