# Supplementary material for: mTORC1 regulates mannose-6-phosphate receptor transport and T-cell vulnerability to regulatory T cells by controlling kinesin KIF13A
Source: Cell Discov. 2017 Apr 25;3:17011–. doi: 10.1038/celldisc.2017.11 (PMC5404257; doi:10.1038/celldisc.2017.11)
Supplement: Supplementary Information [file celldisc201711-s1.pdf]

Supplementary information

mTORC1 regulates mannose-6-phosphate receptor transport and T-cell vulnerability to regulatory T cells by controlling kinesin KIF13A

Khawaja Ashfaque Ahmed[#, †] and Jim Xiang[#, †]

Inventory of supplemental information

Supplementary Figure S1, related to Figure 1

Supplementary Figure S2, related to Figure 1

Supplementary Figure S3, related to Figure 6

Supplementary information

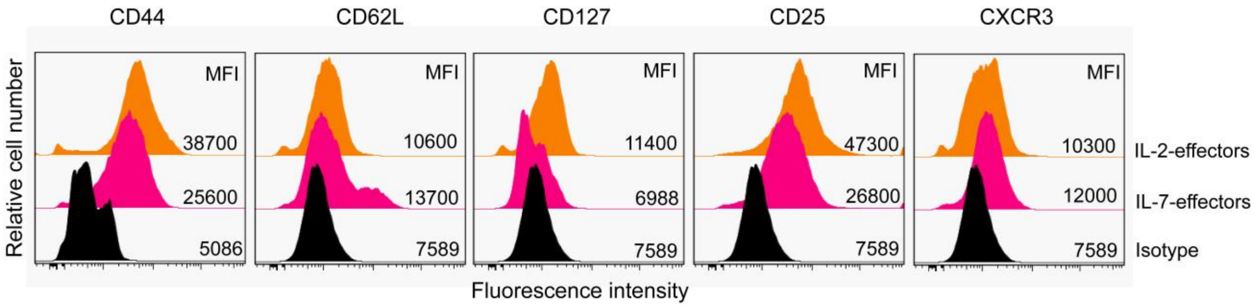

Supplementary Figure S1. Cell-surface receptors and migration markers expression in IL-2 and IL-7 effectors. OT-I CD8$^+$ cells (CD45.1 or CD45.2 congenic) were activated with OVA peptide in the presence of IL-2 (100 U/mL) for 3 days and after washing subsequently cultured for another 2 days in either IL-2 (100 U/mL) or IL-7 (10 ng/mL) to generate IL-2 (CD45.1) and IL-7 (CD45.2) effector CD8$^+$T cells, respectively. Then IL-2 and IL-7 effectors were surface stained for cytokine receptors, adhesion molecules and migration markers. Cells were analyzed by flow cytometry (CytoFLEX, Beckman Coulter). Data are representative of at least two independent experiments (n=6). MFI, mean fluorescence intensity.

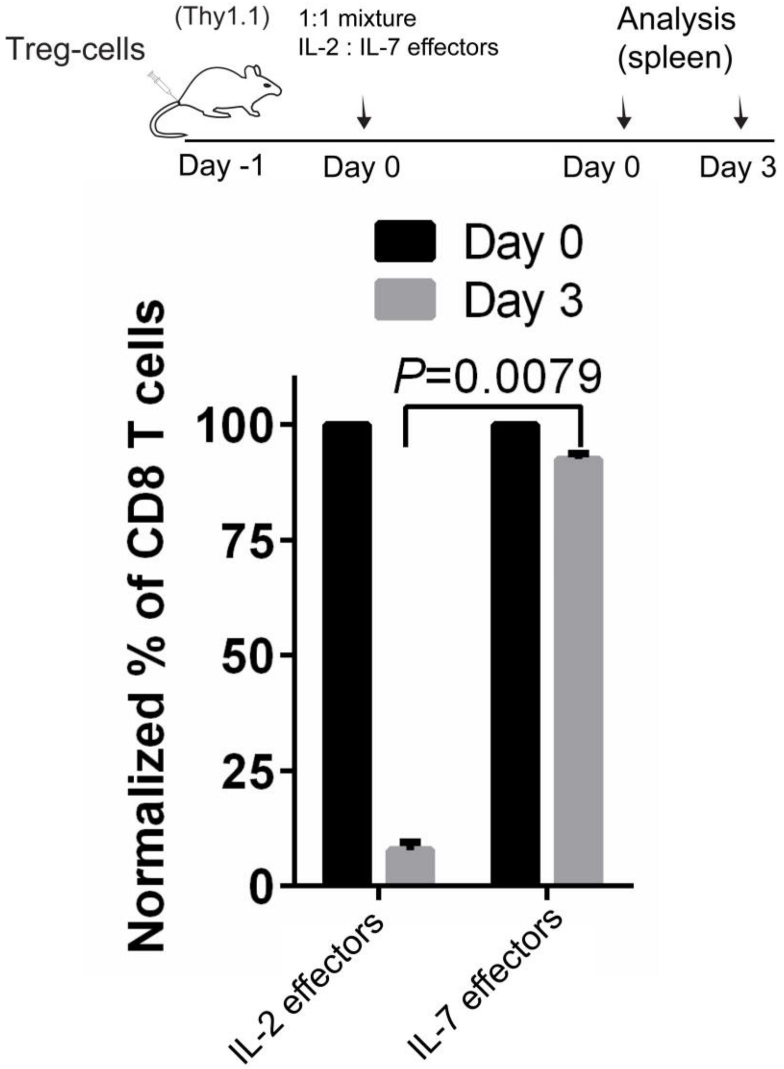

Supplementary Figure S2. IL-2- but not IL-7-stimulated T-cells are preferentially eliminated *in vivo*. OT-I CD8$^+$ cells (CD45.1 or CD45.2 congenic) were activated with OVA peptide in the presence of IL-2 (100 U/mL) for 3 days and after washing subsequently cultured for another 2 days in either IL-2 (100 U/mL) or IL-7 (10 ng/mL) to generate IL-2 (CD45.1) and IL-7 (CD45.2) effector CD8$^+$T cells, respectively. Then 1:1 mixture of IL-2 and IL-7 effectors was injected into Thy1.1 recipient mice (n=6) that had received T_{reg}-cells 1-day before. Splenocytes were analyzed by flow cytometry to count recovered CD8$^+$T cells. Data were analyzed by student *t* test. $P< 0.05$ was considered significant.

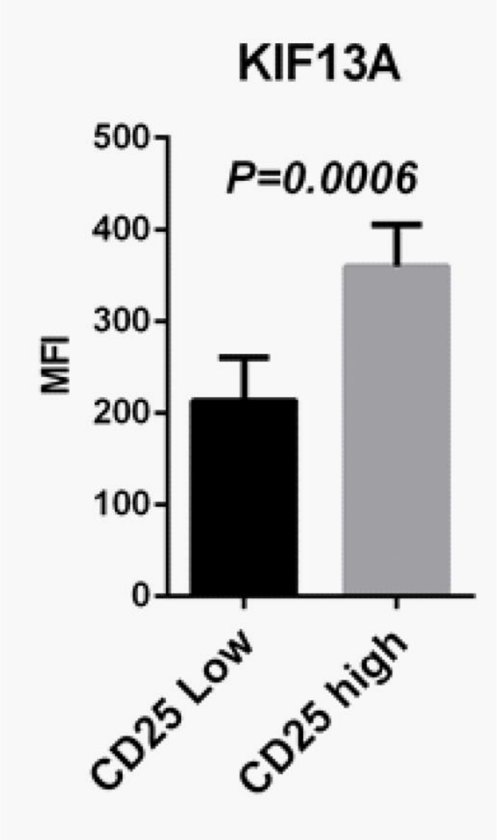

Supplementary Figure S3. CD8$^+$T cells with up-regulated IL-2Rα (CD25) expressed significantly higher levels of KIF13A during LmOVA infection. OT-I CD8$^+$T cells (5,000), CD45.2 congenic, were transferred into recipient (CD45.1), and next day infected with LmOVA (2500 cfu/mice, i.v. injection). On day 5 post-infection, spleens were collected, and intracellular KIF13A was analyzed gating on OVA-specific CD8$^+$ T cells expressing CD25^{high} or CD25^{low}. Results are mean ± S.D (error bars). Data were analyzed by student *t* test. $P < 0.05$ was considered significant. Data are representative of at least two independent experiments (n=6). MFI, mean fluorescence intensity.
